# Extraction of Pharmacokinetic Evidence of Drug–Drug Interactions from the Literature

**DOI:** 10.1371/journal.pone.0122199

**Published:** 2015-05-11

**Authors:** Artemy Kolchinsky, Anália Lourenço, Heng-Yi Wu, Lang Li, Luis M. Rocha

**Affiliations:** 1 School of Informatics and Computing, Indiana University, Bloomington, IN, USA; 2 Instituto Gulbenkian de Ciência, Oeiras, Portugal; 3 ESEI: Escuela Superior de Ingeniería Informática, University of Vigo, Edificio Politécnico, Campus Universitario As Lagoas s/n 32004, Ourense, Spain; 4 CEB—Centre of Biological Engineering, University of Minho, Campus de Gualtar, 4710-057 Braga, Portugal; 5 Center for Computational Biology and Bioinformatics and Department of Medical & Molecular Genetics, Indiana University School of Medicine, Indianapolis, IN, USA; National Center for Biotechnology Information, UNITED STATES

## Abstract

Drug-drug interaction (DDI) is a major cause of morbidity and mortality and a subject of intense scientific interest. Biomedical literature mining can aid DDI research by extracting evidence for large numbers of potential interactions from published literature and clinical databases. Though DDI is investigated in domains ranging in scale from intracellular biochemistry to human populations, literature mining has not been used to extract specific *types of experimental evidence*, which are reported differently for distinct experimental goals. We focus on *pharmacokinetic evidence* for DDI, essential for identifying causal mechanisms of putative interactions and as input for further pharmacological and pharmacoepidemiology investigations. We used manually curated corpora of PubMed abstracts and annotated sentences to evaluate the efficacy of literature mining on two tasks: first, identifying PubMed abstracts containing pharmacokinetic evidence of DDIs; second, extracting sentences containing such evidence from abstracts. We implemented a text mining pipeline and evaluated it using several linear classifiers and a variety of feature transforms. The most important textual features in the abstract and sentence classification tasks were analyzed. We also investigated the performance benefits of using features derived from PubMed metadata fields, various publicly available named entity recognizers, and pharmacokinetic dictionaries. Several classifiers performed very well in distinguishing relevant and irrelevant abstracts (reaching F1≈0.93, MCC≈0.74, iAUC≈0.99) and sentences (F1≈0.76, MCC≈0.65, iAUC≈0.83). We found that word bigram features were important for achieving optimal classifier performance and that features derived from Medical Subject Headings (MeSH) terms significantly improved abstract classification. We also found that some drug-related named entity recognition tools and dictionaries led to slight but significant improvements, especially in classification of evidence sentences. Based on our thorough analysis of classifiers and feature transforms and the high classification performance achieved, we demonstrate that literature mining can aid DDI discovery by supporting automatic extraction of specific types of experimental evidence.

## Introduction

Drug-drug interaction (DDI) is one of the major causes of adverse drug reaction (ADR) and a threat to public health. Pharmaco-epidemiology studies [1] and recent National Health Statistics Report publications [2, 3] indicate that each year an estimated 195,000 hospitalizations and 74,000 emergency room visits are the result of DDI in the United States alone [4]. DDI has been implicated in nearly 3% of all hospital admissions [5] and 4.8% of admissions among the elderly [1] and is a common consequence of medical error, representing 3% to 5% of all inpatient medication errors [6]. With increasing rates of polypharmacy, which refers to the use of multiple medications or more medications than are clinically indicated [7], the incidence of DDI will likely increase in the coming years.

Researchers link molecular mechanisms underlying DDI to their clinical consequences through three types of studies: *in vitro*, *in vivo*, and clinical [8–10]. *In vitro* pharmacology experiments use intact cells (e.g. hepatocytes), microsomal protein fractions, or recombinant systems to investigate molecular interaction mechanisms within the cell (i.e. metabolic, transport- or target-based). *In vivo* studies evaluate whether such interactions impact drug exposure in humans. Finally, clinical studies use a population-based approach and large electronic medical record databases to investigate the contribution of DDI to drug efficacy and ADR.

Automated biomedical literature mining (BLM) methods offer a promising approach for uncovering evidence of possible DDI in published literature and clinical databases [11]. BLM is a biomedical informatics methodology that holds the promise of tapping into the biomedical collective knowledge [12] by extracting information from large-scale literature repositories and by integrating information scattered across various domain-specific databases and ontologies [13–15]. It has been used for knowledge discovery in many biomedical domains, including extraction of protein-protein interactions [16, 17], protein structure prediction [18], identification of genomic locations associated with cancer [19], and mining drug targets [20]. In the domain of DDI, putative interactions uncovered by BLM can serve as targets for subsequent investigation by *in vitro* pharmacological methods as well as *in vivo* and clinical studies [11].

BLM has previously been used for DDI information extraction [21–26], as overviewed by the literature on recent DDI challenges [27–29] and *Pacific Symposium on Biocomputing* sessions [30, 31]. However, much remains to be done in automatic extraction of *experimental evidence of DDI* from text. Importantly, experimental evidence of DDI is reported differently for the different types of studies described above. For instance, *in vivo* pharmacokinetic experiments report parameters such as the ‘area under the concentration-time curve’, while clinical studies may instead report population-level statistics of adverse drug reactions. It is important for BLM pipelines to be able to identify these different kinds of evidence independently.

To address this situation, we demonstrate the use BLM for reliable extraction of *pharmacokinetic* *evidence* for DDI from reports of *in vitro* and *in vivo* experiments. Pharmacokinetic experimental evidence refers to measures of pharmacokinetic parameters such as the inhibition constant (Ki), the 50% inhibitory concentration (IC50), and the area under the plasma concentration-time curve (AUCR). Such evidence is particularly important in identifying or dismissing causal mechanisms behind DDIs and in providing support for putative DDIs extracted from mining patient records, where biases and confounds in reporting often give rise to non-causal correlations [32]. In order to pursue the goal of using BLM to uncover pharmacokinetic DDI evidence, a collaboration was developed between Rocha’s lab, working on literature mining, and Li’s lab, working on pharmacokinetics. Though this work is focused on pharmacokinetic evidence, in subsequent studies we will approach other types of DDI evidence (e.g. clinical evidence).

Our approach is different from previous BLM approaches to DDI information extraction [21–26, 28] because our ultimate goal is not to identify interacting drugs themselves but rather abstracts and sentences containing a *specific type* of *evidence of drug interaction*. Existing DDI-extraction methods and corpora—including those evaluated under the DDI Extraction challenges [27–29, 33, 34]—are not well suited for this task because they do not attempt to extract *experimental evidence* of drug interactions, nor specifically label distinct kinds of evidence. For instance, the DDI Extraction challenge ‘11 [33] used a corpus of several hundred documents from DrugBank [35], but interacting drug pairs were annotated without regard for the presence of experimental evidence. More recently, the DDI Extraction challenge ‘13 [34] provided a corpus annotated with pharmacokinetic and pharmacodynamic interactions [29], but the goal of the text mining task was the extraction and classification of interacting drug pairs, not the extraction of the experimental evidence of interactions. Other related work has used DrugBank data for large-scale extraction of drug-gene and drug-drug relationships [22, 36], and for predicting DDI using a drug-drug network based on phenotypic, therapeutic, chemical, and genomic feature similarity [37], but neither study aimed to identify or extract specific kinds experimental evidence of DDI.

We have previously shown that BLM can be used for automatic extraction of numerical pharmacokinetics (PK) parameters from the literature [38]. However, that work was not oriented specifically toward the extraction of evidence of DDI. Recently, we reported high performance in a preliminary work on automatically classifying PubMed abstracts that contain pharmacokinetic evidence of DDI [39] (details below). Because identifying relevant abstracts is only a first step in the process of extracting pharmacokinetic evidence of DDI, in this work we consider both the problem of identifying abstracts containing pharmacokinetic evidence of DDI and that of extracting from abstracts sentences that contain this specific kind of evidence. In addition to evidence sentence extraction, we also provide a new assessment of abstract classification using an updated version of a separately published corpus [26], leading to substantially better classification performance than reported in our preliminary study [39]. The updated corpus is described below and is publicly available. Finally, we provide a new comparison of classifiers, a new evaluation methodology using permutation-based significance tests and Principal Component Analysis (PCA) [40] of feature weights, and a detailed study of the benefits of including features derived from PubMed metadata, named entity recognition tools and specialized dictionaries.

We created abstract and sentence corpora using annotation criteria for identifying pharmacokinetic evidence of DDI. We consider positive (indicating the presence of interactions) *and* negative (indicating the absence) DDI evidence as relevant (see “Materials and Methods” section), since both provide important information about possible DDI. Because the criteria considered here are different from those used in previously available DDI corpora, our results are not directly comparable to other BLM approaches to DDI. Therefore, we pursued a thorough evaluation of the performance of different types of classifiers, feature transforms, and normalization techniques. For both abstract and sentence classification tasks we tested several linear classifiers: logistic regression, support vector machines (SVM), binomial Naive Bayes, linear discriminant analysis, and a modification of the Variable Trigonometric Threshold (VTT) classifier, previously developed by Rocha’s lab and found to perform well on protein-protein interaction text mining tasks [12, 41, 42]. As we describe in the results and discussion sections, classifiers fall into two main classes based on whether or not they take into account feature covariances. In addition, we compared different feature transform methods, including normalization techniques such as ‘Term Frequency, Inverse Document Frequency’ (TFIDF) and dimensionality reduction based on Principle Component Analysis (PCA). We also compared performance when including features generated by several Named Entity Recognition (NER) tools and specialized dictionaries.

In the experiments reported, our goal is to measure the quality of automated methods in identifying pharmacokinetic evidence of DDIs reported in the literature. More generally, we seek to demonstrate that literature mining can be successful in automatically extracting experimental evidence of interactions as part of DDI workflows. We show that many classifier configurations achieve high performance on this task, demonstrating the robustness and efficacy of BLM on extracting pharmacokinetic evidence of DDI.

## Materials and Methods

**Fig 1:**
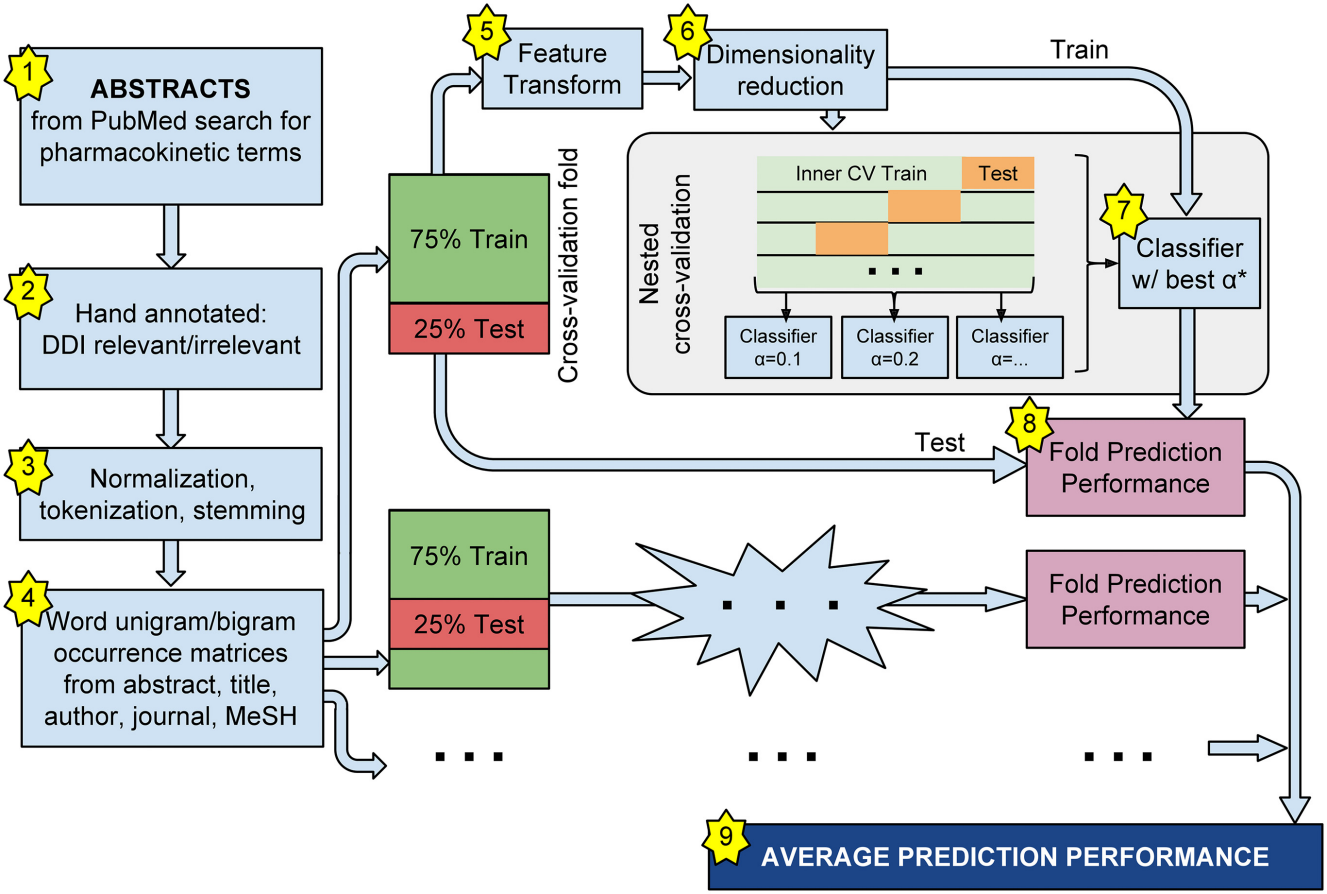
Literature mining pipeline. The basic steps of the literature mining pipeline include selection of corpus documents, hand-labeling of ground truth assignments, extraction and normalization of textual features, and computation of unigram/bigram occurrences matrices. Cross-validation folds are used to estimate generalization performance of classifier and feature transform configurations, while nested (inner) cross-validation folds are used to choose classifier hyperparameters.

The following sections describe the methods used in our literature mining pipeline. Its basic steps are visually diagrammed in Fig 1. They include the selection of corpus documents, hand-labeling of ground truth assignments, extraction and normalization of textual features, and computation of unigram/bigram occurrences matrices. Cross-validation folds are used to estimate generalization performance of classifier and feature transform configurations, while nested (inner) cross-validation folds are used to choose classifier hyperparameters. The software consisted of custom Python scripts unless otherwise noted.

### Abstract Corpus

For the training corpus, Li’s lab selected 1203 pharmacokinetics-related abstracts by searching PubMed using terms from a previously developed ontology for PK pharmacokinetic parameters [38]. Therefore, all retrieved articles describe and contain some form of pharmacokinetic evidence, though not necessarily of DDI. We kept *in vitro* studies but removed any animal *in vivo* studies. Abstracts were labeled according to the following criteria: abstracts that reported *the presence or absence of drug interaction supported by explicit experimental evidence of pharmacokinetic parameter data* were labeled as *DDI-relevant* (909 abstracts) while the rest were labeled as *DDI-irrelevant* (294 abstracts). DDI-relevance was established regardless of whether the relevant enzymes were presented or not. Importantly, the concept of DDI-relevance employed here updates the criteria used in a previous preliminary study [39]. Interactions between a drug and food, fruit, smoking, alcohol, and natural products are now classified as drug interactions because their pharmacokinetics studies are designed similarly. For the same reason, studies dealing with interactions between drug metabolites (instead of parent compounds) are now also considered relevant, as well as studies reporting inhibition of induction of a drug on a drug metabolism enzyme or drug transporter. Classification was done by three graduate students with M.S. degrees and one postdoctoral annotator; any inter-annotator conflicts were checked by a Pharm D. and an M.D. scientist with extensive pharmacological training. The corpus is publicly available as “Pharmacokinetics DDI-Relevant Abstracts V0” in [43] (see also [26]).

We extracted textual features from PubMed article title and abstract text fields as well as the following metadata fields: the author names, the journal title, the Medical Subject Heading (MeSH) terms, the ‘registry number/EC number’ (RN) field, and the ‘secondary source’ field (SI) (the latter two fields contain identification codes for relevant chemical and biological substances). For each PubMed entry, the content of the above fields was tokenized, processed by Porter stemming [44], and converted into textual features (unigrams and, in certain runs, bigrams). Strings of numbers were converted into ‘#’, short textual features (with length of less than 2 characters) and infrequent features (that occurred in less than 2 documents) were omitted. Author names, journal titles, substance names, and MeSH terms were treated as single textual tokens.

The corpus was represented as binary term-document occurrence matrices. We evaluated classification performance under two different conditions: in the first—referred to as ‘unigram runs’—only word unigram features were used; in the second—referred to as ‘bigram runs’—word bigram features were used in addition to unigram features. Bigram runs included a much larger number of parameters (i.e. the bigram feature coefficients) that needed to be estimated from training data, which can potentially increase generalization error arising from increased model complexity [45]. Testing the classifiers exclusively with unigram features as well as with both unigram and bigram features evaluated whether the class information provided by bigrams outweighed their cost in complexity.

### Sentence Corpus

The evidence sentence task consisted in identifying those sentences within a PubMed abstract that reported experimental evidence for the presence or absence of a specific DDI. For this purpose, Li’s group developed a training corpus of 4600 sentences extracted from 428 PubMed abstracts. All abstracts contained (positive or negative) pharmacokinetic evidence of DDIs. Sentences were manually labeled as DDI-relevant (1396 sentences) if they *explicitly mentioned pharmacokinetic evidence for the presence or absence of drug-drug interactions*, and as DDI-irrelevant (3204 sentences) otherwise. The same pre-processing and annotation procedures were followed for the sentence corpus as for the abstract corpus (see section “Abstract Corpus”). This corpus is publicly available as “Deep Annotated PK Corpus V1” in [43] (see also [26]).

### Classifiers

Six different linear classifiers were tested:

1. *VTT*: a simplified, angle-domain version of the *Variable Trigonometric Threshold* Classifier, previously developed in Rocha’s lab [12, 41, 42]. Given a document vector **x** = <*x_1_*, …, *x_K_*> with features (i.e. dimensions) indexed by *i*, the separating hyperplane is defined as
  $$\begin{array}{c} \sum_{i}\varphi_{i}x_{i}-\lambda =0 \end{array}$$
  Here, *λ* is a threshold (bias) and *φ_i_* is the ‘angle’ of feature *i* in binary class space:
  $$\begin{array}{c} \varphi_{i}=\arctan\frac{p_{i}}{n_{i}}-\frac{\pi }{4} \end{array}$$
  where *p_i_* is the probability of occurrence of feature *i* in relevant-class documents and *n_i_* is the probability of occurrence of feature *i* in irrelevant-class documents. The threshold parameter *l* is chosen so that a neutral ‘pseudo-document’ defined by *x_i_* = (*p_i_*+*n_i_*)/2 falls exactly onto the separating hyperplane.
  The full version of VTT, which includes additional parameters to account for named entity occurrences and which we have previously used in protein-protein interaction classification, is evaluated in combination with various NER tools in section “Impact of NER and PubMed metadata on abstract classification” below. VTT performs best on sparse, positive datasets; for this reason, we do not evaluate it on dense dimensionality-reduced datasets. Notice that in previous work, we used a different version of VTT with a cross-validated threshold parameter; its performance on the tasks was very similar, and is reported in the Supporting Information as the ‘VTTcv’ classifier (section 1 and 2 in S1 Text).
2. *SVM*: a linear *Support Vector Machine* with a cross-validated regularization parameter (implemented using the sklearn [46] library’s interface to the LIBLINEAR package [47]).
3. *Logistic regression* classifier with a cross-validated regularization parameter (also implemented using sklearn’s interface to LIBLINEAR).
4. *Naive Bayes* classifier with smoothing provided by a Beta-distributed prior with a cross-validated concentration parameter.
5. *LDA*: a regularized *Linear Discriminant Analysis* classifier, following [48]. Singular value decomposition (SVD), a dimensionality reduction technique, is first used to reduce any rank-deficiency, after which the covariance matrix is shrunk toward a diagonal, equal-variance structured estimate. The shrinkage parameter is determined by cross-validation.
6. *dLDA*: a ‘diagonal’ LDA, where only the diagonal entries of the covariance matrix are estimated and the off-diagonal entries are set to 0. A cross-validated parameter determines shrinkage toward a diagonal, equal-variance estimate. This classifier can offer a more robust estimate of feature variances; it is equivalent to a Naive Bayes classifier with Gaussian features [49].

Generally, linear classifiers fall into one of two types. Classifiers of the first type—sometimes called ‘naive’ in the literature, which in our case include VTT, dLDA, and Naive Bayes—learn feature weights without considering feature covariances. While covariance information can be useful for distinguishing classes, naive classifiers often perform well with small amounts of training data, when covariances are difficult to estimate accurately. Classifiers of the second type—which we refer to as ‘non-naive’, and which in our case included SVM, LDA, and Logistic Regression—do consider feature covariances (often in combination with regularization techniques to smooth covariance estimates) and can achieve superior performance when provided with sufficient training data.

### Feature Transforms

For both unigram and bigram runs, we evaluated classification performance on several transforms of the document matrices:

1. No transform: raw binary occurrence matrices (see section “Abstract Corpus”).
2. IDF: occurrences of feature *i* were transformed to its Inverse Document Frequency (IDF) value: $\text{idf}\left(i\right)=\log\frac{N}{c_{i}+1}$, where *c_i_* is the total number of occurrences of feature *i* among all documents. This reduced the influence of common features on classification.
3. TFIDF: the Term Frequency, Inverse Document Frequency transform (TFIDF); same as above, but subsequently divided by the total number of features that occur in each document. This reduced the impact of document size differences.
4. Normalization: the non-transformed, IDF, and TFIDF document matrices underwent a length-normalization transform, where each document vector was inversely scaled by its L2 norm. L2 normalization has been argued to be important for good SVM performance [50].
5. PCA: The above matrices were run through a Principal Component Analysis (PCA) dimensionality reduction step. Projections onto the first 100, 200, 400, 600, 800, and 1000 components were tested.

Feature transforms can improve classification performance by making the surfaces that separate documents in different classes more linear and by decreasing the weight of non-discriminating features. PCA, on the other hand, reduces the number of parameters that need to be estimated from training data. If class membership information is contained in the subspace spanned by the largest principal components, then this kind of dimensionality reduction can improve generalization performance by reducing noise and model complexity.

### Performance evaluation

The abstract and sentence corpora described above were used both for training classifiers and for estimating generalization performance on out-of-sample documents. In order to estimate out-of-sample performance, we used the following cross-validation procedure for each possible classifier and feature transform:

1. Each corpus was randomly partitioned into 4 document folds (75%–25% splits). This was repeated 4 times, yielding 16 *outer folds*. All classifiers and transforms were evaluated using the same partitions.
2. For each fold, the 75% split was treated as the ‘training’ split and the 25% split was treated as the ‘testing split’. If a feature transform was used, it was applied to both splits but was computed using statistics (such as IDF or principal components) from the training split. Finally, classifiers were trained on the training split and evaluated based on their prediction performance on the testing split.
3. Measures of classification performance (see below) on the testing split were collected. The 16 sets of performance measures were averaged to produce an estimate of generalization performance.

Because training and testing documents are always separated, for each cross-validation fold the above procedure is equivalent to calculating performance on an independent testing corpus.

Except for VTT, the classifiers listed in section “Classifiers” used cross-validated regularization parameters. These parameters were not chosen using cross-validation on the outer folds because this would lead to a biased estimate of out-of-sample performance. Instead, regularization parameters were chosen using nested cross-validation within each of the 75% blocks of the above outer folds:

1. The 75%-block was itself partitioned into 4 folds (75%–25% splits of the outer 75% block). This is repeated 4 times, producing a total of 16 *inner folds* for each outer fold training split.
2. Over a range of values of the cross-validated parameter, the procedure described in step 2 above was used, but now applied to the 75%/25% splits of each *inner fold*. Mean performance on inner fold testing splits were measured using the Matthews Correlation Coefficient [51] (MCC), which is particularly well-suited for the unbalanced scenarios of our corpora [52].
3. The parameter value giving the highest mean MCC was chosen as the regularization parameter value for training the classifier in the outer fold.

We evaluated the performance of the classifiers using three different measures: the balanced F1 score (the harmonic mean of precision and recall), the iAUC or ‘area under the interpolated precision/recall curve’ [53], and the MCC. In addition, we computed and reported the rank product of these three measures (RP3) as a single inclusive metric of classification performance. The RP3 measure provides a well-rounded assessment of classifier performance, as it combines the ranking of the different individual measures [12, 41].

For displaying results, we focus primarily on the iAUC measure (in cases where only plots of iAUC performance are provided, F1 and MCC plots are found in the Supporting Information, S1 Text). iAUC does not depend on predicted class assignments but rather on the ranking of test set documents according to classifier confidence scores from most relevant to most irrelevant. iAUC offers three major advantages as a measure of classification performance. First, it provides a more comprehensive measure of classifier performance because it evaluates the entire ranking of documents, as opposed to just class assignments. Second, iAUC is less sensitive to variation driven by random-sampling differences in the training corpus, which may lead to fluctuations in the class assignments of low confidence documents and, correspondingly, high variability in measures such as F1 and MCC. Finally, it is more relevant in a frequently-encountered situation where a human practitioner uses a BLM pipeline to retrieve only the most relevant documents (which should have high positive-class confidence scores) or to identify likely-to-be-misclassified documents (which should have low confidence scores).

Both the abstract and sentence classification tasks are characterized by imbalanced datasets, with more relevant-class abstracts and more irrelevant-class sentences respectively. For simplicity, and because we are primarily concerned how ranking performance (as measured by iAUC) changes between different machine learning configurations on the same dataset, we do not perform resampling or re-weighting of training items. We also report MCC values, a measure which is known to be stable in the face of unbalanced classes [52].

The performance of a classifier and feature transform configuration varies both due to random sampling of folds and due to the inherent performance bias of the configuration over the entire distribution of folds. Since we are only interested in the latter, observed performance differences between pairs of configurations were tested for statistical significance using a non-parametric paired-sample permutation test. First, the assignments of performance scores for each of the 16 outer folds were permuted between the two classifier/transform configurations under consideration. For each of the 2^16^ possible permutations, the difference in across-fold mean performance was calculated; this formed the distribution of performance differences under the null hypothesis that the two configurations have equal performance. Finally, the *p*-value was computed as the probability (one- or two-tailed, as indicated) of observing a difference under the null hypothesis distribution equal to or greater than the actual difference.

## Results

### Abstract classification performance

**Fig 2:**
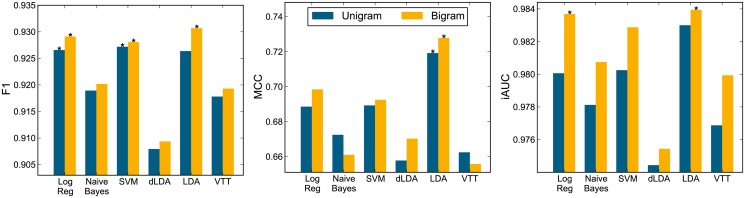
Classification performance on abstracts. Performance for both unigram and bigram runs on non-transformed features. Left: F1 measure. Middle: MCC measure. Right: iAUC measure. The best classifier configuration, and configurations not significantly different (*p*>0.05, one-tailed test) from it, marked with asterisk ‘*’.

Fig 2 shows classifier performance on the abstract task for the unigram and bigram runs with no feature transform applied. The best classifier configuration, as well as those configurations not significantly different from the best (*p*>0.05, one-tailed test), are marked with an asterisk. In addition, the performance results, ranks, and the rank-product (RP3) measure are reported in Table 1. The best classifier achieves F1≈0.93, iAUC≈0.98, MCC≈0.73, which constitutes a substantial and significant improvement over our previous preliminary results reported in [39], where we had reached F1≈0.8, iAUC≈0.88, MCC≈0.6 (notice that these performance values would be well below the lowest reported levels in Fig 2). This demonstrates that the corpus used in this work—which is more carefully curated and now also considers interactions between drugs and food, fruit, smoking, alcohol, and natural products to be relevant (details in “Materials and Methods Section”)—improves the classification of abstracts with pharmacokinetic evidence of DDI. The levels of performance achieved are excellent when compared to similar abstract classification tasks in other biomedical domains. For instance, in the BioCreative Challenge III, considered one of the premier forums for assessment of text mining methods, the best classifiers of abstracts with Protein-Protein Interaction yielded performances of F1≈0.61, iAUC≈0.68, MCC≈0.55 [17]. Naturally, our results are not directly comparable to results obtained on different corpora and on a different problem; rather, these numbers provide guidance on what is typically considered good results in biomedical article classification.

**Table 1:** Classification performance on abstracts. Performance for both unigram and bigram runs on non-transformed features according to F1, MCC, and iAUC performance measures. The rank of the classifiers according to each measure is reported in parenthesis in the respective column. Classifiers are ordered according to the rank product (RP3) of the three measures (last column).

**Classifier**	**Type**	**F1**	**MCC**	**iAUC**	**RP3**
LDA	Bigram	.931 (1)	.728 (1)	.984 (1)	1
Log Reg	Bigram	.929 (2)	.698 (3)	.984 (1)	6
SVM	Bigram	.928 (3)	.693 (4)	.983 (3)	36
LDA	Unigram	.926 (6)	.719 (2)	.983 (3)	36
Log Reg	Unigram	.927 (4)	.689 (5)	.980 (6)	120
SVM	Unigram	.927 (4)	.689 (5)	.980 (6)	120
Naive Bayes	Bigram	.920 (7)	.661 (10)	.981 (5)	350
Naive Bayes	Unigram	.919 (8)	.672 (7)	.978 (9)	504
VTT	Bigram	.919 (8)	.656 (12)	.980 (6)	576
VTT	Unigram	.918 (10)	.662 (9)	.977 (10)	900
dLDA	Bigram	.909 (11)	.670 (8)	.975 (11)	968
dLDA	Unigram	.908 (12)	.658 (11)	.974 (12)	1584

For each classifier, the inclusion of bigram features improved performance according to the RP3 measure. The best classifier according to all measures was LDA using bigrams. The performance of this classifier was significantly better than all others for the MCC, but not significantly better that Logistic Regression according to iAUC, and not significantly better that Logistic Regression and SVM according to F1 score. According to RP3, these three classifiers using bigrams yield the best performance. Naive Bayes, VTT, and dLDA—classifiers that make a ‘naive’ independence assumption about features (see “Materials and Methods” section)—performed below the top three. However, the performance levels they achieved are still quite high, which indicates that such simple classifiers are also capable of classifying documents with pharmacokinetic DDI evidence in our corpus. The in-house VTT classifier is the only classifier among these that does not use cross-validated parameters; when used with NER features and cross-validated parameters (the configuration for which it was originally designed [12, 41, 42]), its performance improved (see below).

#### Feature Transforms and Dimensionality Reduction

The different feature transforms and PCA-based dimensionality reductions (section “Materials and Methods”) significantly improved performance for several classifiers, though they could not beat the performance of the best non-transformed classifier. Details are provided in Supporting Information (section 1.1 in S1 Text). To summarize, according to most measures only dLDA and SVM improved performance significantly with either an IDF or TFIDF transform plus L2 normalization and dimensionality reduction (top *n* principal components). For instance, the best iAUC for SVM (0.984) occurs with a dimensionality reduction to the top 800 principal components and no feature transform; this is a significant improvement over the no-transform, no dimensionality reduction SVM classifier reported in Table 1 and Fig 2, but not a significant improvement over the overall best classifiers reported there (LDA and Logistic regression). The dLDA classifier significantly improves its iAUC performance with almost all feature transform and dimensionality reduction combinations, but not above that of the top performing classifiers. We conclude that feature transforms and dimensionality reduction does not lead to the best classification performance on the abstract task.

#### Pharmacokinetics DDI Features in abstract classification

We looked at which textual features play the largest role in the abstract classification task. A linear classifier separates document classes with a hyperplane defined by a set of feature coefficients. The impact of a feature on classification is quantified by the sign and amplitude of its hyperplane coefficient. A feature with a large positive coefficient contributes strongly to a document’s propensity to be classified as relevant, while a feature with a large negative coefficient contributes strongly to a document’s propensity to be classified as irrelevant. In Table 2, we show the top 20 most distinctive features of the relevant and irrelevant classes in the abstract task, as chosen in the bigrams runs by the LDA classifier (left) and Logistic Regression classifier (right), the two top-performing classifiers in this task according to the RP3 measure (see Table 1). Notice that textual features are stemmed.

**Table 2:** Top 20 relevant and irrelevant abstract features. The stemmed textual features most discriminative of relevant and irrelevant classes on the abstract task, as chosen by two of the top-performing classifiers according to the RP3 measure: LDA with bigrams (left) and Logistic Regression with bigrams (right).

**LDA (Bigram)**		**Logistic Regression (Bigram)**	
**Relevant**	**Irrelevant**	**Relevant**	**Irrelevant**
*MeSH:Drug Interactions*	area	*MeSH:Drug Interactions*	area
interact	rate	interact	rate
inhibit	differ	inhibit	differ
interact between	polymorph	interact between	*MeSH:Reference Values*
oral	activ	oral	activ
day	genotyp	decreas	that the
decreas	higher	mg	conclus
receiv	patient with	*Substance:Enzyme Inhibitors*	clearanc of
mg	conclus	receiv	higher
increas	to the	auc	patient
auc	*Substance:Hydrocarb. Hydroxylas.*	determin	*MeSH:Female*
inhibitor	that the	treatment	patient with
*MeSH:Male*	lower	day	*MeSH:Injections, Intravenous*
treatment	patient	inhibitor	polymorph
chang	allel	chang	genotyp
increas the	among	dure	*MeSH:Phenotype*
dure	extens	alon	among
on the	*MeSH:Female*	*MeSH:Male*	healthi subject
alon	of the	administ	*MeSH:Half-life*
combin	analysi	chang in	lower

Some of the most relevant features come from MeSH term metadata (such as the MeSH term *Drug interactions*) and terms that explicitly indicate interactions (‘interact’, ‘inhibit’, ‘interact between’, ‘decreas’, ‘increas’). Other relevant terms deal with administration protocols and study design (‘oral’, ‘day’, ‘receiv’, ‘mg’, ‘treatment’, ‘alon’, ‘combin’). Some of the irrelevant features concern genetics terminology (‘allel’, ‘genotyp’, ‘polymorph’, and MeSH term *Phenotype*), indicating that the irrelevant class was enriched with genetics or pharmacogenetics vocabulary. Several generic biomedical terms (such as ‘patient’, ‘healthi subject’, ‘higher’) terms are also highly irrelevant. In addition, highly irrelevant features also contain some non-DDI-specific pharmacokinetic terms (for example, ‘area’, ‘rate’, ‘clearance of’), which is not surprising given that both relevant and irrelevant articles were drawn from pharmacokinetics-related literature. One surprising result is the observation that while the MeSH term *Mal*e is one of the top relevant features, the MeSH term *Female* is one of the top irrelevant features. We have no explanation for the cause of this gender imbalance since the corpus was built from automatic searches to PubMed without any gender-specific query terms.

Further analysis of highly relevant and irrelevant features across all classifiers and feature transforms was performed and reported in the Supporting Information (section 1.2 in S1 Text). We quantified and plotted the contribution of standardized coefficients [54] of different features and show the most positively and negatively loaded features for different classifier and transform configurations. Top textual features obtained from all classifiers include additional terms falling under the categories described above, with features derived from PubMed metadata (MeSH, chemical substances) also appearing among both the most relevant and irrelevant sets. Other relevant MeSH terms, besides *Drug Interactions*, include *Cimetidine/pharmacology*, *Cross-Over Studies*, *Enzyme Inhibitors/PK*, *Kinetics*, and *Proton Pump Inhibitors*. Additionally, a PubMed author entry corresponding to a prominent researcher in the pharmacokinetics DDI field (‘PJ Neuvonen’) appears as highly relevant, as well as three substances from the RN field (see also section “Impact of NER and PubMed metadata on abstract classification” below): *Cimetidine*, *Enzyme Inhibitors*, and *Proton Pump Inhibitors*. For the irrelevant set, additional MeSH (*Anti-Ulcer Agents/adm&dos*; *Injections, Intravenous*; *Phenotype*; *Protein Binding*; *Reference Values*) and Substance terms also appear (*Anti-ulcer agents*; *Hydrocarb. Hydroxylas*). The Supporting Information S1 Text contains details of the analysis and lists of features. It also shows the results of a Principal Component Analysis of feature weight coefficients chosen by different classifiers.

#### Impact of NER and PubMed metadata on abstract classification

We have previously demonstrated improved classification performance on protein-protein interaction BLM tasks by supplementing textual features (such as the word unigram and bigram occurrences) with features built using *Named Entity Recognition* (NER) and domain-specific *dictionary* tools [12, 41, 42]. To test if similar techniques are useful in the DDI domain, we counted mentions of named biochemical species (e.g. proteins, compounds and drugs) and concepts (e.g. pharmacokinetic terms) in each document and then included these counts as document features in addition to the bigram and unigram textual features. Counts were extracted using biomedical-specific NER extraction tools and dictionaries, with dictionary matches identified by internally-developed software. A preliminary study of the impact of NER/Dictionary features was reported in [39] using a previous less-refined DDI corpus. Here, in addition to using the more fine-tuned corpus (see “Methods and Data” section), we study the impact of PubMed metadata features on classification performance. We also provide a new comprehensive analysis of the performance impact of including features from several publicly-available NER and metadata resources:

- OSCAR4 [55]: NER tool for chemical species, reaction names, enzymes, chemical prefixes and adjectives.
- ABNER [56]: NER tool for genes, proteins, cell lines and cell types.
- BICEPP [57]: NER tool for clinical characteristics associated with drugs.
- *DrugBank* database [58]: a dictionary list of drug names
- *Dictionaries* provided by Li’s lab. *i-CYPS*: cytochrome P450 [CYP] protein names, a group of enzymes centrally involved in drug metabolism; *i-PkParams*: terms relevant to pharmacokinetic parameters and studies; *i-Transporters*: proteins involved in transport; *i-Drugs*: Food and Drug Administration’s drug names. The dictionaries are available for download from [43].

For each of these NER tools and dictionaries, we counted the number of occurrences of any of its entities/entries in a given abstract. These counts were treated as any other feature for SVM, Logistic Regression, diagonal LDA, and LDA classifiers. Naive Bayes was omitted since NER count features are non-binary. VTT incorporates NER features via a modified separating hyperplane equation:

$$\begin{array}{c} \sum_{i}\varphi_{i}x_{i}-\sum_{j}\frac{\beta_{j}-c_{j}}{\beta_{j}}-\lambda =0 \end{array}$$

where *x_i_* represents the occurrence of textual feature *i*, *φ_i_* and *λ* are textual feature and bias parameters as described in section “Classifiers”, *c_j_* is the count of NER/Dictionary feature from resource *j*, and *β_j_* is a weight for resource *j*, which is chosen by cross-validation.

**Fig 3:**
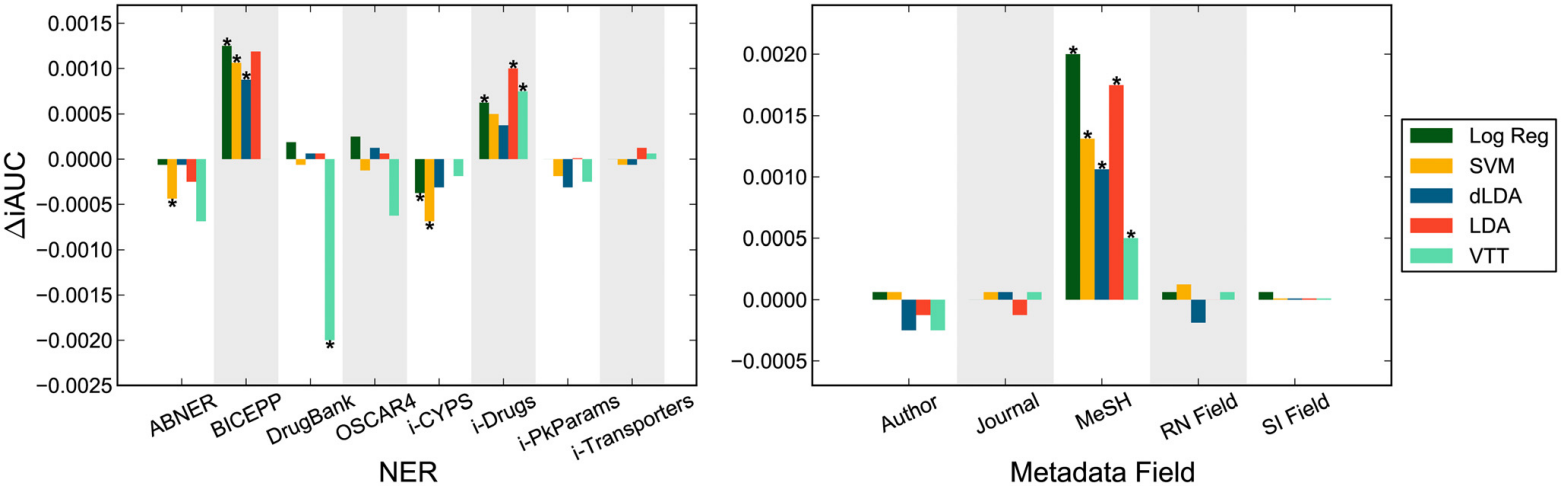
Performance impact of abstract NER and metadata features. Left: Relative changes in iAUC scores on non-transformed bigram runs in combination with different NER/Dictionary features. Significant changes (*p*<0.05, two-tailed test) in performance over the respective classifiers without NER features are indicated with asterisk ‘*’. Right: Relative changes in iAUC when features from a given PubMed metadata field are included versus omitted (while including features from the other 4 metadata fields). Significant changes (*p*<0.05, two-tailed test) in performance are indicated with asterisk ‘*’.

In Fig 3 (left), we plot the relative iAUC changes over the respective classifiers without NER/Dictionary count features (results for MCC and F1 in Supporting Information; section 1.3 in S1 Text). Significant performance changes are indicated with an asterisk (*p*<0.05, two-tailed test). Some NER/Dictionary features improved performance significantly for several classifiers. However, the inclusion of two dictionary features (*DrugBank*, and *i-CYPS*) actually decreased performance significantly for several classifiers, suggesting that these features contain little class information and instead contribute to over-fitting. Table 3 lists performance for configurations in which NER and dictionary features gave a significant performance increase for at least one of the three measures (F1, MCC, or iAUC), along with best classifier performance using only textual features (bigram runs). The BICEPP tool consistently yielded the best improvement for every classifier tested, followed by the *i-Drugs* dictionary. The OSCAR4 tool also significantly improved the performance of the VTT classifier (especially for the MCC measure as shown in Supporting Information, S1 Text). With the inclusion of NER and dictionary features, the overall top classifiers (LDA and Logistic Regression), significantly improved their performance, now reaching F1≈0.93, MCC≈0.74, iAUC≈0.99. Among the set of naive classifiers, VTT improved performance significantly with the inclusion of NER features, ranking above the other naive classifiers according to the RP3 measure.

**Table 3:** Abstract classification performance using NER features. Performance of the best classifiers when specific NER and dictionary features are added; original (bigram runs) classifiers also listed with no NER features (indicated by -). F1, MCC, and iAUC performance measures are listed; the rank of the classifiers according to each measure is reported in parenthesis in the respective column. Classifiers are ordered according to the rank product (RP3) of the three measures (last column).

**Classifier**	**NER**	**F1**	**MCC**	**iAUC**	**RP3**
LDA	BICEPP	.933 (2)	.737 (1)	.985 (1)	2
LDA	*i-Drugs*	.934 (1)	.736 (2)	.985 (1)	2
Log Reg	BICEPP	.933 (2)	.714 (3)	.985 (1)	8
Log Reg	*i-Drugs*	.930 (6)	.700 (6)	.985 (1)	36
LDA	–	.931 (5)	.728 (3)	.984 (5)	75
SVM	BICEPP	.932 (4)	.710 (5)	.984 (5)	100
Log Reg	–	.929 (8)	.698 (7)	.984 (5)	280
SVM	*i-Drugs*	.930 (6)	.687 (10)	.984 (5)	300
SVM	–	.928 (9)	.693 (8)	.983 (9)	648
VTT	BICEPP	.922 (11)	.692 (9)	.980 (12)	1188
VTT	OSCAR4	.923 (10)	.683 (11)	.979 (14)	1540
VTT	*i-Drugs*	.920 (12)	.670 (14)	.981 (10)	1680
Naive Bayes	–	.920 (12)	.661 (16)	.981 (10)	1920
dLDA	BICEPP	.911 (15)	.680 (12)	.976 (15)	2700
VTT	–	.919 (14)	.656 (17)	.980 (12)	2856
dLDA	*i-Drugs*	.911 (15)	.678 (13)	.975 (16)	2700
dLDA	–	.909 (17)	.670 (14)	.975 (16)	3808

As mentioned, word unigram and bigram features were extracted not only from article abstracts and titles, but also from five PubMed metadata fields: author names, journal titles, MeSH terms, and two fields referring to standardized substance names: the ‘registry number/EC number’ [RN] field and the ‘secondary source’ field [SI]. In fact, some PubMed metadata features were among those most distinguishing of relevant and irrelevant abstracts (for greater detail, see Table 2 and section “Pharmacokinetics DDI Features in abstract classification”, as well Supporting Information; section 1.2 in S1 Text). We tested the impact of PubMed metadata fields on abstract classification performance. In Fig 3 (right), we plot the relative iAUC changes when features from a given PubMed metadata field are included versus omitted (while including features from the other 4 metadata fields). Significant changes (*p*<0.05, two-tailed test) in performance are indicated with an asterisk; results for MCC and F1 can be found in Supporting Information (S1 Text). MeSH terms was the only metadata source whose omission decreased performance significantly. However, the performance increase of including MeSH data is rather small. Therefore, the methodology does not require the availability of human-annotated metadata such as MeSH terms and can still be deployed on recent articles that have not yet been annotated with MeSH terms.

### Evidence sentence extraction performance

**Fig 4:**
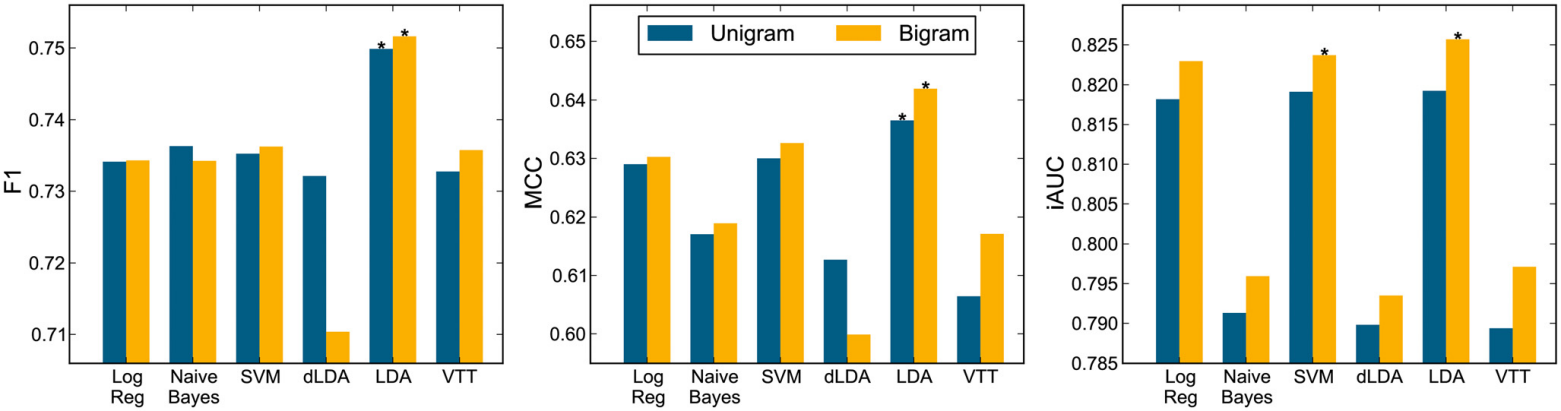
Sentence classification performance. Performance for both unigram and bigram runs on non-transformed features. Left: F1 measure. Middle: MCC measure. Right: iAUC measure. The best classifier configuration, and configurations not significantly different (*p*>0.05, one-tailed test) from it, marked with asterisk ‘*’.

Fig 4 shows classification performance on the sentence task of the unigram and bigram runs without any feature transforms applied, according to F1, MCC, and iAUC measures. The best classifier configuration, as well as those configurations not significantly different from the best (*p*>0.05, one-tailed test), are marked with an asterisk. In addition, the numerical results, ranks, and the rank-product (RP3) measure are reported in Table 4.

**Table 4:** Sentence classification performance. Performance for both unigram and bigram runs on non-transformed features according to F1, MCC, and iAUC performance measures. The rank of the classifiers according to each measure is reported in parenthesis in the respective column. Classifiers are ordered according to the rank product (RP3) of the three measures (last column).

**Classifier**	**Type**	**F1**	**MCC**	**iAUC**	**RP3**
LDA	Bigram	.752 (1)	.642 (1)	.826 (1)	1
LDA	Unigram	.750 (2)	.636 (2)	.819 (4)	16
SVM	Bigram	.736 (3)	.633 (3)	.824 (2)	18
Log Reg	Bigram	.734 (7)	.630 (4)	.823 (3)	84
SVM	Unigram	.735 (6)	.630 (4)	.819 (4)	96
VTT	Bigram	.736 (3)	.617 (8)	.797 (7)	168
Naive Bayes	Unigram	.736 (3)	.617 (8)	.791 (10)	240
Log Reg	Unigram	.734 (7)	.629 (6)	.818 (6)	252
Naive Bayes	Bigram	.734 (7)	.619 (7)	.796 (8)	392
dLDA	Unigram	.732 (11)	.613 (10)	.790 (11)	1210
dLDA	Bigram	.710 (12)	.600 (12)	.794 (9)	1296
VTT	Unigram	.733 (10)	.606 (11)	.789 (12)	1320

As with abstracts, including bigram features tended to improve sentence classification performance. LDA performed best, having the highest RP3 and being the best classifier according to the F1 and MCC measures and one of the two best classifiers (along with SVM) on the iAUC measure. Generally, the classifiers that performed well on the sentence task were those that took into account feature covariances: SVM, Logistic Regression, and LDA. The top classifier (LDA with bigrams) on the evidence sentence task reached performance of F1≈0.75, MCC≈0.64, iAUC≈0.83.

We measured sentence classification performance in combination with different feature transforms and dimensionality reductions (see section 2.1 in S1 Text). In general, the three classifiers that do best on non-transformed features (SVM, Logistic Regression, and LDA) show decreased performance with dimensionality reduction according to all measures, with more extreme dimensionality reduction leading to larger performance decreases. On the other hand, for dLDA (a ‘naive’ classifier that treats features as independent), PCA-based dimensionality reduction—which uses feature covariances to choose optimal projections—led to significant improvements in all measures, with more dimensions giving better performance. These findings indicate that the pattern of feature covariance carries important information about class membership in the sentence task, and that this pattern is distributed across a large number of dimensions. Generally, the LDA classifier achieved the best performance according to all three measures. Its baseline performance according to the iAUC measure was further improved significantly by an IDF-transform, and—according to the F1 measure—by any transform containing an L2 normalization.

In Table 5, we show the top 20 features most distinctive of the relevant and irrelevant classes in the sentence task as chosen by the LDA classifier on bigrams (the top performing classifier according to the RP3 measure; features of other top classifiers are not shown because they were highly similar). Numerical features (indicated by ‘#.#’) were highly indicative of the relevant class, along with expressions of quantitative changes (‘decreas’, ‘increas’) and interaction (‘inhibit’, ‘catalyz’, ‘interact with’) as well as adverbs expressing significance of evidence (‘significantli’). Also highly relevant were features referring to the area under the concentration-time curve (‘auc’), which is often employed in pharmacokinetics to measure differences in drug clearance rates under different experimental conditions. Names of several drugs (‘ketoconazol’, ‘itraconazol’, ‘quiindin’) were relevant in predicting DDI evidence sentences. These drugs are frequently used probe inhibitors for metabolism enzymes CYP3A4/5, CYP3A4/5 and CYP2D6 respectively and are routinely used in drug interaction studies.

**Table 5:** Top 20 relevant and irrelevant sentence features. The most discriminative features of relevant and irrelevant classes in the sentence task, as chosen by the top-performing classifiers according to the RP3 measure: LDA on bigrams.

**LDA (Bigram)**	
**Relevant**	**Irrelevant**
inhibit	day
increas	investig
#.#	determin
ketoconazol	vitro
decreas	evalu
microm	enzym
rifampin	use
format	differ
catalyz	cytochrom p450
auc	studi
significantli	dose
coadministr	examin
itraconazol	measur
quinidin	subject
clearanc	assess
reduc	interact
#.#-fold	compar
show	drug
co-administr	genotyp
interact with	cytochrom

Highly irrelevant features refer to more generic pharmacokinetic or biomedical concepts such as ‘investig’, ‘dose’, ‘enzym’, ‘studi’, etc. Interestingly, some terms that are highly relevant in the abstract task are highly irrelevant in the sentence task (e.g., ‘day’). Notably, the unigram ‘interact’ is highly irrelevant for sentences, whereas the bigram ‘interact with’ is highly relevant. This may be because all sentences in this corpus come from abstracts containing pharmacokinetic DDI evidence (see “Materials and Methods” section). Thus, general administration protocols and drug interaction terms are likely to occur in the abstract as a whole but not necessarily in the evidence sentences that actually report outcomes of the pharmacokinetic drug interaction experiments. Similar patterns are observed in the more extensive analysis provided in Supporting Information (section 2.2 in S1 Text), where relevant and irrelevant features are analyzed across a wide range of classifier and feature transform configurations. There we also show the results of a Principal Component Analysis of feature weight coefficients chosen by different classifiers.

**Fig 5:**
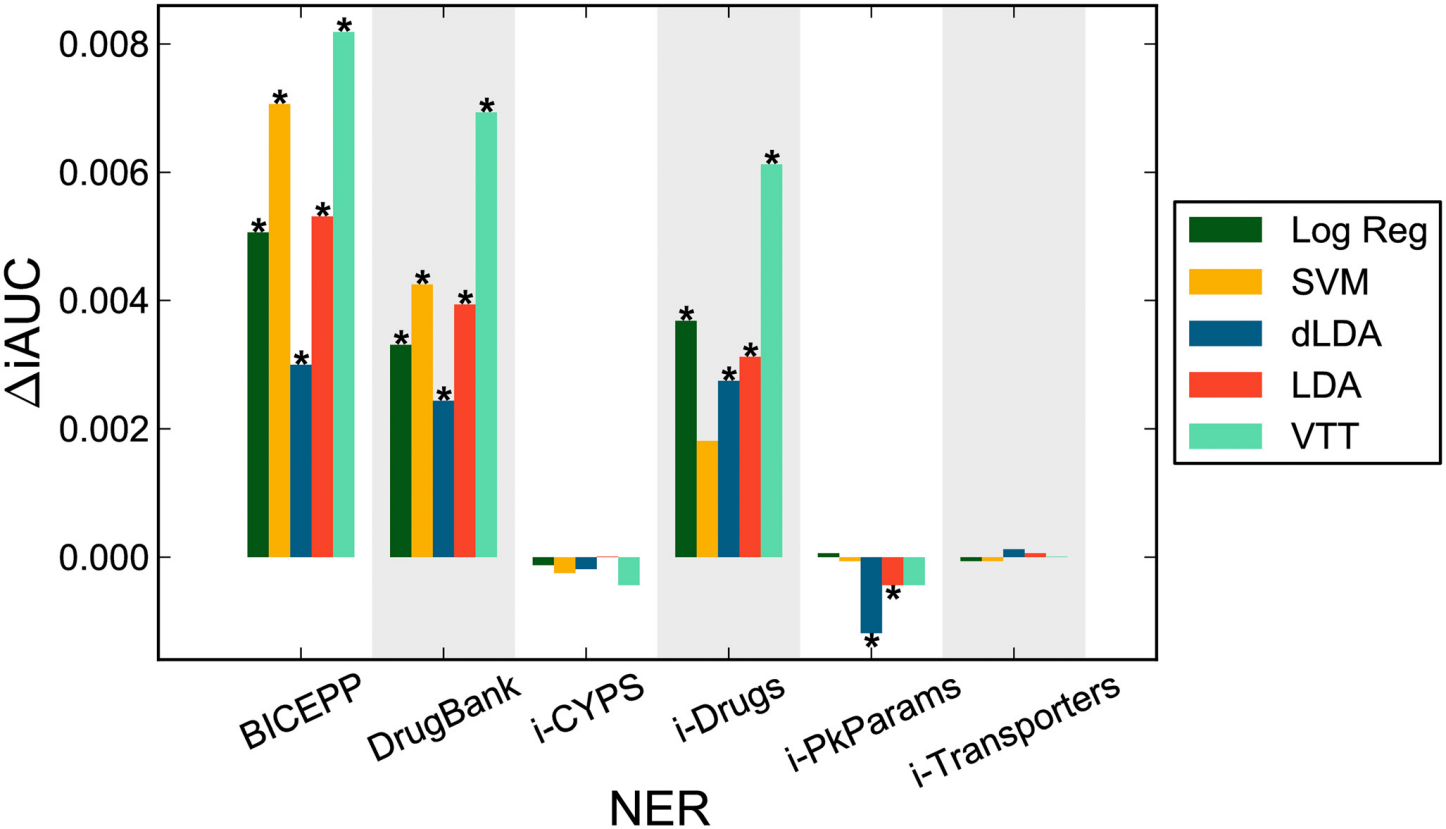
Performance impact of sentence NER features. Relative changes in iAUC scores on sentence bigram runs (without transforms or dimensionality reductions) in combination with different NER features. Significant changes (*p*<0.05, two-tailed test) in performance over respective classifiers without NER features are indicated with asterisk ‘*’.

Finally, we tested the impact of additional features on sentence classification. Though there is no metadata available in the sentence corpus, features from NER tools can still be computed. Six NER features were tested: *BICEPP, DrugBank, i-CYPS, i-Drugs, i-PkParams, i-Transporters* (see section “Impact of NER and PubMed metadata on abstract classification” for details). As before, we counted mentions of named biochemical species and concepts specified by different NER tools in each sentence and then included such counts as sentence features in addition to the bigram and unigram textual features. Fig 5 shows relative iAUC changes when features from each of these NER tools were included. Significant improvements (*p*<0.05, two-tailed test) above the corresponding classifier’s performance without NER features are indicated by an asterisk; performance according to MCC and F1 measures is shown in Supporting Information (section 2.3 of S1 Text). Notice that Naive Bayes was omitted since NER count features are non-binary.

As in the abstract task, a few NER/Dictionary features improved performance for several classifiers. The iAUC scores of nearly all classifiers were significantly improved by three NER features: BICEPP, *DrugBank*, and our internally developed *i-Drugs* dictionary. These three features represent counts of drugs names, showing that drug name counts are helpful for classifying sentences as DDI-relevant vs. DDI-irrelevant. Use of features from the BICEPP tool yielded the largest improvement for every classifier. Table 6 lists the performance according to all measures for classifiers using the BICEPP features; also listed are the corresponding best classifiers using only textual features (bigram runs). The overall top classifiers (LDA and SVM) showed significantly improved performance with the inclusion of these NER features, reaching F1≈0.76, MCC≈0.65, iAUC≈0.83. In addition, VTT performance improved significantly for all three measures with the inclusion of NER features. Here VTT with bigrams performs better than other naive classifiers, as expected given that this classifier was designed specifically to handle such NER features [12, 41, 42]. In contrast, dLDA (another naive classifier) did not benefit much from the inclusion of NER features.

**Table 6:** Sentence classification performance with NER features. Performance of different sentence classifiers with the count features obtained via the BICEPP NER tool; also listed are the corresponding best classifiers using only textual features (bigram runs; indicated by -). F1, MCC, and iAUC performance measures are listed; the rank of the classifiers according to each measure is reported in parenthesis in the respective column. Classifiers are ordered according to the rank product (RP3) of the three measures (last column).

**Classifier**	**Type**	**F1**	**MCC**	**iAUC**	**RP3**
LDA	BICEPP	.757 (1)	.650 (1)	.831 (1)	1
SVM	BICEPP	.741 (4)	.639 (3)	.831 (1)	12
LDA	–	.752 (2)	.642 (2)	.826 (4)	16
Log Reg	BICEPP	.738 (5)	.634 (4)	.828 (3)	60
VTT	BICEPP	.742 (3)	.629 (7)	.805 (7)	147
SVM	–	.736 (6)	.633 (5)	.824 (5)	150
Log Reg	–	.734 (8)	.630 (6)	.823 (6)	288
VTT	–	.736 (6)	.617 (8)	.797 (8)	432
Naive Bayes	–	.734 (8)	.619 (8)	.796 (10)	640
dLDA	BICEPP	.711 (10)	.603 (10)	.797 (8)	800
dLDA	–	.710 (11)	.600 (11)	.794 (11)	1331

## Discussion

We have demonstrated that current BLM methods for text classification can reliably identify PubMed abstracts containing pharmacokinetic evidence of drug-drug interactions, as well as extract specific sentences that mention such evidence from relevant abstracts. The performance reached on a corpus of carefully annotated pharmacokinetics literature is quite high for both abstract classification (reaching F1≈0.93, MCC≈0.74, iAUC≈0.99) and evidence sentence extraction (F1≈0.76, MCC≈0.65, iAUC≈0.83). To explore the capability of BLM in the pharmacokinetics DDI context, where there are no existing directly-relevant corpora or experiments, we pursued a thorough comparison of the performance of several linear classifiers using different combinations of unigrams, bigrams, PubMed metadata, and NER features. We also tested the effects of applying feature transforms and dimensionality reduction.

From a classification performance perspective, some results are noteworthy: in terms of textual features, bigrams in combination with unigrams performed significantly better than unigrams alone. However, performance in unigram versus bigram runs for the same classifier differed by no more than one percent for iAUC and MCC. Thus, while bigram features did contain some additional information about class membership, the amount of this information was not large.

In our experiments, feature transforms and PCA-based dimensionality reduction significantly improved performance for several classifiers (especially “naive” classifiers such as dLDA, which assume feature independence), but did not significantly improve the overall best performance. We also found that a sophisticated version of the LDA classifier dominated performance in both the abstract and sentence tasks. This classifier used SVD to eliminate rank-deficiency in the feature occurrence matrices and performed shrinkage of the feature covariance matrix for regularization (see “Materials and Methods”).

From the drug-interaction domain perspective, feature analysis in the abstract task revealed that pharmacokinetic DDI evidence in the literature is highly correlated with terms that explicitly indicate interaction (including MeSH terms), enzyme inhibitors (including substance names via the RN metadata field in PubMed), DDI administration protocols, and study design. At the sentence level, drug interaction evidence from a pharmacokinetics perspective is highly correlated with terms that express experimental results, such as numerical values, measures of drug clearance, expressions of quantitative changes, as well as adverbs expressing significance of evidence. Feature analysis at the abstract level also revealed that lack of DDI evidence in the pharmacokinetics literature (irrelevant class) is highly correlated with some terms from PubMed metadata fields, as well as those pertaining to genomic or general medical terminology. At the sentence level, sentences in relevant abstracts but without DDI evidence tend to include terminology relevant to pharmacokinetics protocols, as well as more generic interaction discourse or biomedical concepts.

Since many important features came from PubMed metadata fields, we looked at changes in iAUC scores when features from different PubMed metadata fields were omitted. We found that only the omission of MeSH terms significantly affected abstract classification performance. Nonetheless, while statistically significant, the drop in performance was rather small (affecting only millesimals of the iAUC, iAUC≈0.98 without), indicating that abstract classification does not depend strongly on the inclusion of MeSH term features. This is an important consideration since MeSH terms may not be immediately added to publications, with statistics indicating that only 50% of citations are annotated within 60 days of inclusion in PubMed [59]. Therefore, classification and evidence extraction from brand new articles should not rely on such metadata.

We also tested the effect of including features extracted using named entity recognition (NER) and dictionary tools, namely those for detecting possibly-relevant chemical, genomic, metabolomic, drug, and pharmacokinetic entities. Generally, dictionaries like BICEPP, *i-Drugs*, and DrugBank, which counted the number of times drug names appeared, significantly improved performance for several classifiers on both the abstract classification and evidence sentence extraction tasks (an exception to this was the lack of improvement on abstracts when including DrugBank features, an effect that needs further investigation). Nonetheless, as for MESH term features in abstract classification, the resulting performance increases were modest, even if statistically significant. This again demonstrates that relevant-class information can be extracted from abstracts and sentences using solely the statistics of unigram and bigram textual features.

Notably, relevant and irrelevant documents and sentences both derive from the pharmacokinetics literature and therefore share similar feature statistics. This makes distinguishing between them a nontrivial text classification problem, though also a more practically relevant one (e.g. for a researcher who needs to automatically label potentially relevant documents retrieved from PubMed). Nonetheless, several classifiers reached high performance; for example, the abstract ranking performance (iAUC≈0.99) has little room for further improvement, though the classification performance—while high for this type of problem—can still be improved.

We observed that many different pipeline configurations reached near-optimal performance. Even though some performance differences between configurations were statistically significant, they were small. For instance, iAUC differences between best and worst classifiers varied by no more than 1 percent in the abstract task and 5 percent in the sentence task. This demonstrates that classification performance in our experiments was robust to the classifier utilized, and that a BLM pipeline for this problem would do similarly well independently of classifier chosen. In particular, while “non-naive” classifiers (which consider feature covariances) performed better than naive classifiers, the latter are still capable of competitive performance. These results suggest a fundamental limit on the amount of statistical signal present in the labels and feature distributions of the corpora as extractable by linear classifiers. However, it is worth noticing that an analysis of both abstract- and sentence-trained feature weight coefficients shows systematic differences between weights selected by naive and non-naive classifiers (see Supporting Information, S1 Text), indicating that different classifiers emphasize distinct semantic features. Furthermore, it is possible that performance could be improved by the use of non-linear classifiers or features produced by more finely DDI-tuned NER tools, relation extraction or NLP methods, or other sophisticated feature-generation techniques. Indeed, the larger performance variation observed in the sentence task suggests that sentence extraction performance may improve with larger amounts of training data (which would permit better estimates of feature covariances).

It is not trivial to compare our performance results with those previously reported in the literature. First, there is no gold standard for DDI evidence sentence extraction, especially for a specific evidence-type such as pharmacokinetics. Second, most sentence extraction tasks in the biomedical domain involve extraction of passages which can contain several sentences (e.g. the protein-protein interaction subtask in Biocreative II) or passages relevant for a set of specific targets (e.g. Gene Ontology annotations for specific gene names in Biocreative I [60] and IV [61]). Due to these difficulties, the performance on those tasks has been comparatively low, e.g. in BioCreative IV the best F1 score in the gene ontology evidence extraction task was 0.27 [61] (in Biocreative II, due to possible overlap and multiple accepted passages, the preferred performance measure was the mean reciprocal rank which reached 0.87 [12, 62]). Considering that our performance on the sentence task is higher than what is typically reported for the abstract classification in the biomedical domain (e.g. PPI abstract classification in the BioCreative Challenge III reached F1≈0.61, MCC≈0.55, iAUC≈0.68 [17]), the classifiers trained on our sentence corpus reached a very good level of performance, indicating that the corpus is well annotated and that the task is highly feasible. Given the performance of our approach in extracting pharmacokinetic evidence, the classification methodology and associated corpus may be useful in the previously explored task of extracting interacting drug pairs from the literature. For example, it may be more effective to first identify DDI sentences containing specific types of evidence and then extract the interacting drug names from them, using automated methods or human expertise tailored to that specific type of DDI evidence.

To conclude, we provide a thorough report of the capability of linear classifiers to automatically extract pharmacokinetics evidence of DDI from an abstract- and sentence-level annotated corpus. Given the high performance observed on both abstract and sentence classification for all classifiers, including the simplest ones, we conclude that under realistic classification scenarios automatic BLM techniques can identify PubMed abstracts reporting DDI backed by pharmacokinetic evidence, as well as extract evidence sentences from relevant abstracts. These results are important because pharmacokinetic evidence can be essential in identifying causal mechanics of putative DDI and as input for further pharmacological and pharmaco-epidemiology investigation. More generally, our work shows that BLM can be safely included in DDI discovery pipelines where attention to distinct types of evidence is necessary. In future work, we intend to use our methodology to mine large corpora for both pharmacokinetic and other types of DDI experimental evidence. Such evidence can help fill knowledge gaps that exist in the DDI domain, with the ultimate goal of reducing the incidence of adverse drug reactions and contributing to the development of alternative safe treatments.

## Supporting Information

S1 Text Additional performance analysis. Detailed performance measures on both abstract and sentence tasks, including when dimensionality reduction methods and NER and metadata features are used. In addition, the most relevant and irrelevant features for different classifiers and feature transform configuration are provided for both tasks. Feature weight coefficients for different classifiers are compared using PCA.
(PDF)
