# Supplementary material for: Extraction of Pharmacokinetic Evidence of Drug–Drug Interactions from the Literature
Source: PLoS One. 2015 May 11;10(5):e0122199. doi: 10.1371/journal.pone.0122199 (PMC4427505; doi:10.1371/journal.pone.0122199)

Extraction of Pharmacokinetic Evidence of Drug-drug Interactions from the Literature: SUPPORTING INFORMATION

Artemy Kolchinsky, Anália Lourenço, Heng-Yi Wu, Lang Li, Luis M. Rocha

1 Abstract performance

The following table lists classification performance according to F1, MCC, iAUC, and Accuracy measures for different classifiers on unigram and bigram abstract runs. It includes the VTTcv classifier (VTT with a cross-validated threshold), which is not discussed in the main article.

Classifier	Type	F1	MCC	iAUC	Accuracy
Log Reg	Bigram	.929	.698	.984	.891
Log Reg	Unigram	.927	.689	.980	.888
Naive Bayes	Bigram	.920	.661	.981	.878
Naive Bayes	Unigram	.919	.672	.978	.878
SVM	Bigram	.928	.693	.983	.890
SVM	Unigram	.927	.689	.980	.888
VTTcv	Bigram	.916	.697	.980	.877
VTTcv	Unigram	.911	.663	.977	.868
dLDA	Bigram	.909	.670	.975	.868
dLDA	Unigram	.908	.658	.974	.865
LDA	Bigram	.931	.728	.984	.897
LDA	Unigram	.926	.719	.983	.891
VTTcv	Bigram	.916	.697	.980	.877
VTTcv	Unigram	.911	.663	.977	.868
VTT	Bigram	.919	.656	.980	.876
VTT	Unigram	.918	.662	.977	.876

1.1 Abstract performance: Feature transforms and dimensionality reduction

The following charts show iAUC, F1, and MCC performance on abstract bigram runs when feature transforms and dimensionality reductions are applied. Stars indicate configurations that performed significantly better ($p<0.05$, one-tailed test) than the no-transform, no-dimensionality-reduction configuration of the same classifier (indicated with larger circles). Naive Bayes was not tested since it is only applicable to binary data, and VTT was only tested on the sparse transforms (i.e., without PCA-based dimensionality reduction).

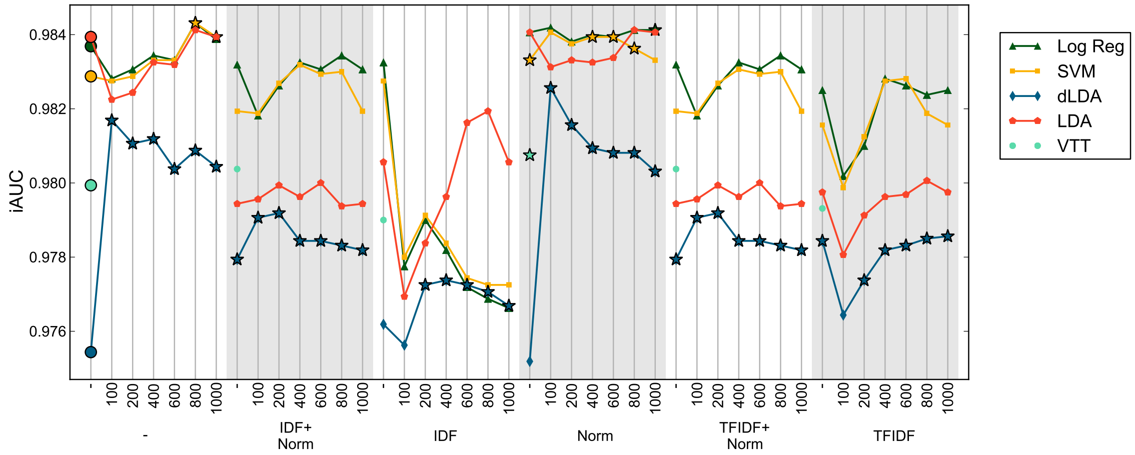

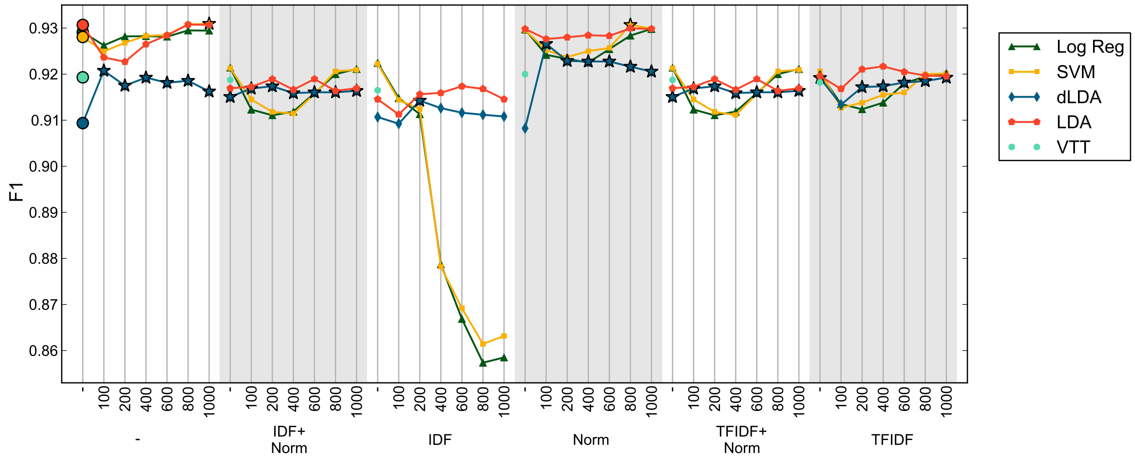

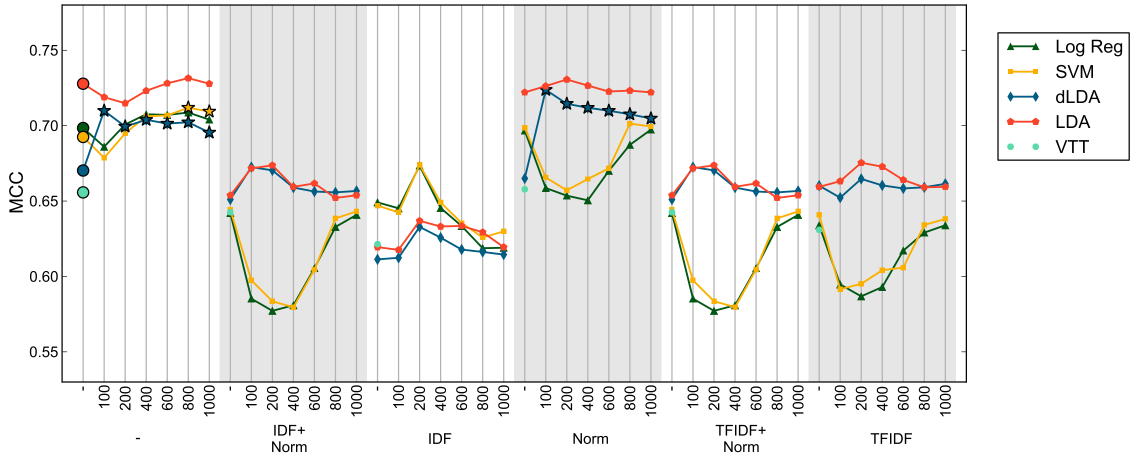

1.2 Abstract Performance: Most relevant and irrelevant features

A linear classifier separates classes using a hyperplane defined by a set of feature coefficients. The impact of a given feature on classification is naturally quantified by the sign and amplitude of its hyperplane coefficient. The increase of the value of a feature with a large positive (negative) coefficient produces a large increase in a document's propensity to be classified as relevant (irrelevant). Coefficients for different features are made comparable by an appropriate normalization: we multiply each feature's coefficient by the standard deviation of the feature's values in the training data, producing what is referred to as a 'standardized coefficient' in the linear regression literature. For the abstract bigram runs, the following figure shows the ranks of the most relevant and the reverse rank of the most irrelevant features. *RELEVANT FEATURES* includes any feature whose standardized coefficient was among the top 5 most positive standardized coefficients for any transform/classifier combination, while *IRRELEVANT FEATURES* includes any feature whose standardized coefficient was among the top 5 most negative standardized coefficients for any transform/classifier combination. Transforms are organized in the vertical columns, while classifiers are distinguished by color and marker style. For relevant (irrelevant) features, markers are positioned according to their rank (reverse rank) among the most positive (negative) features for a given classifier and transform combination.

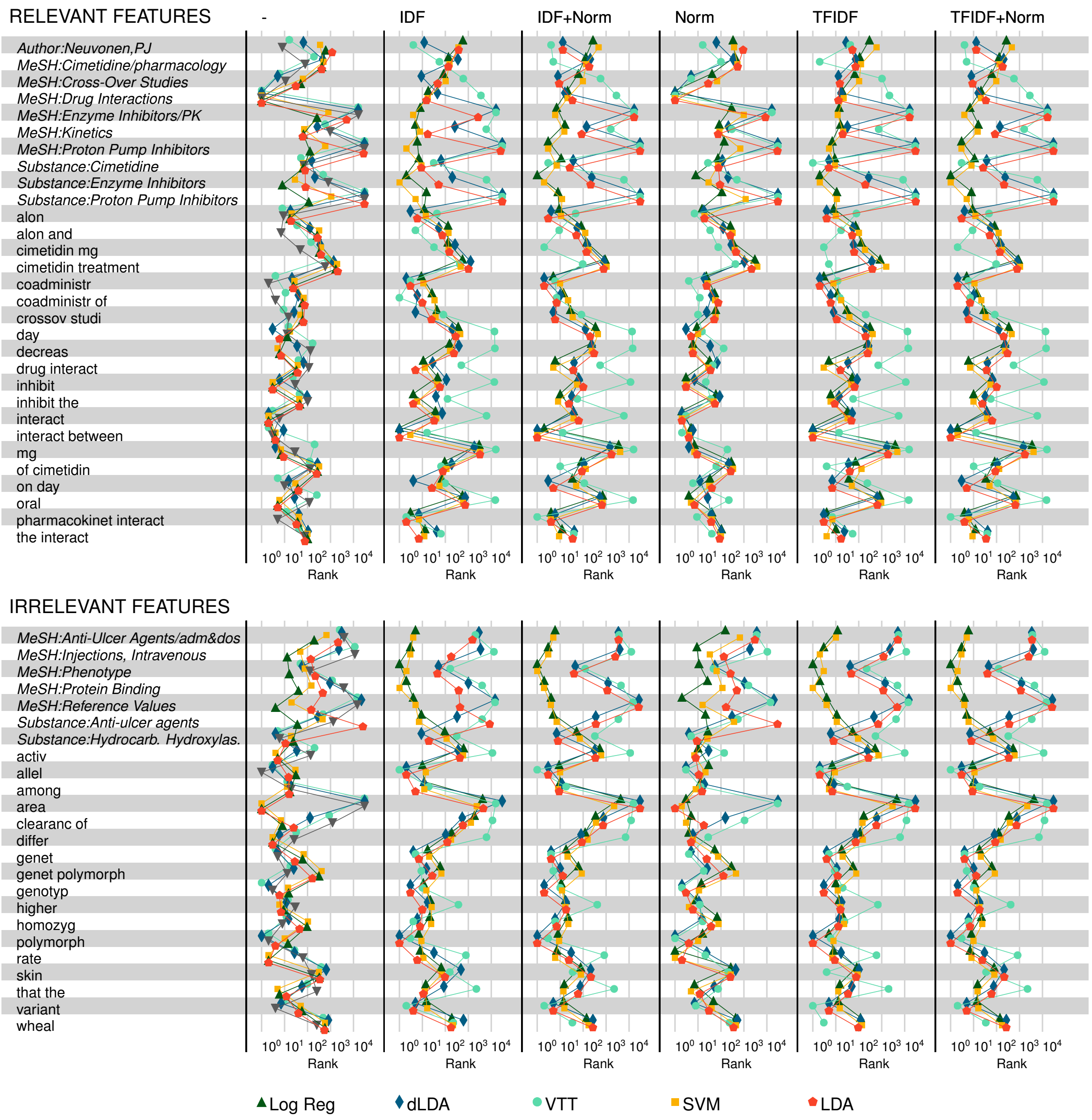

To further compare the importance that different classifier/transform combinations gave to different features, we performed principal component analysis on a matrix composed of the hyperplane coefficients of all such combinations. The following figure shows each transform-classifier hyperplane in terms of its loading on the first two principal components (PCs). This projection separates classifiers that use feature covariance information (LDA, SVM, and Logistic Regression) and those that don't (Naive Bayes, dLDA, VTT). It also groups configurations according to feature transforms, with configurations that included IDF-like transforms clustering separately from those that used no transforms or a simple L2-normalization. In general, SVM and Logistic Regression produce very similar feature loadings, likely due to the fact that they optimize similar cost functions during training.

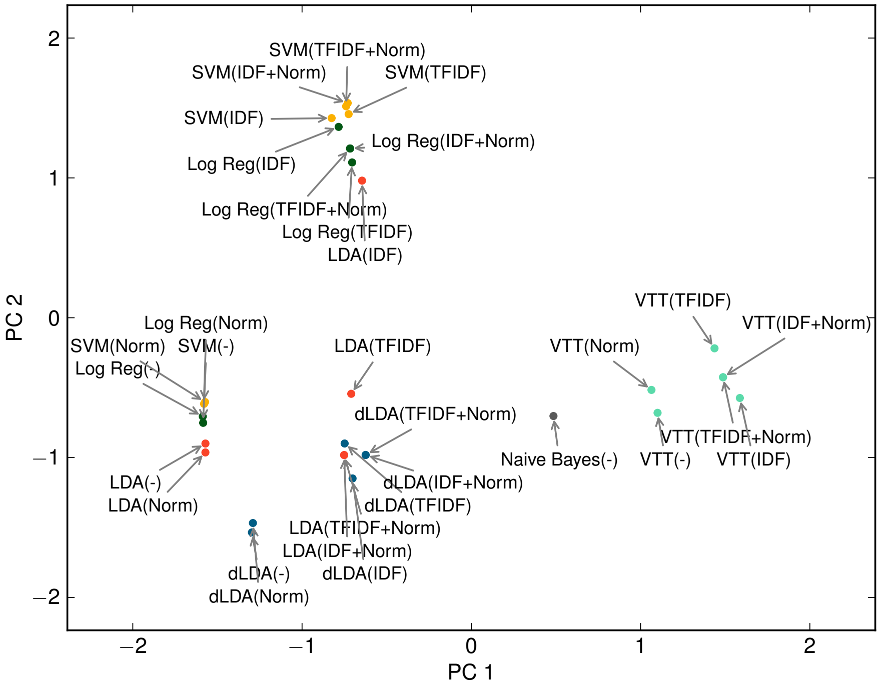

1.3 Abstract Performance: NER and Metadata Features

The following figures plot the relative changes in F1 and MCC performance when including vs. not-including metadata and NER-derived features on abstract bigram runs with feature transforms. Significant changes ($p<0.05$, two-tailed test) are indicated by asterisks. For metadata, changes in performance are measured while still including features from the other 4 metadata fields.

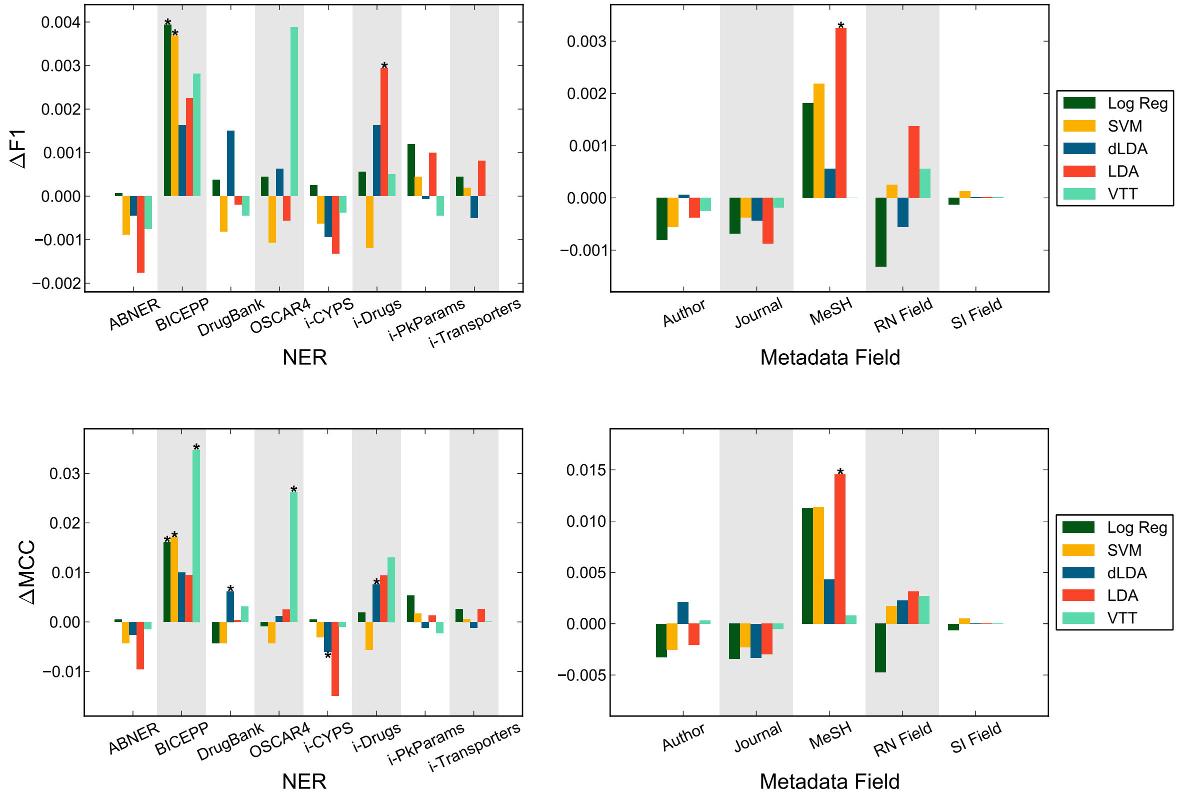

2 Sentence performance

The following table lists classification performance according to F1, MCC, iAUC, and Accuracy measures for different classifiers on unigram and bigram sentence runs. It includes the VTTcv classifier (VTT with a cross-validated threshold), which is not discussed in the main article.

Classifier	Type	F1	MCC	iAUC	Accuracy
Log Reg	Bigram	.734	.630	.823	.848
Log Reg	Unigram	.734	.629	.818	.847
Naive Bayes	Unigram	.736	.617	.791	.835
Naive Bayes	Bigram	.734	.619	.796	.839
SVM	Unigram	.735	.630	.819	.847
SVM	Bigram	.736	.633	.824	.848
VTTcv	Bigram	.733	.608	.797	.822
VTTcv	Unigram	.729	.608	.789	.831
dLDA	Bigram	.710	.600	.794	.836
dLDA	Unigram	.732	.613	.790	.834
LDA	Bigram	.752	.642	.826	.848
LDA	Unigram	.750	.636	.819	.843
VTTcv	Bigram	.733	.608	.797	.822
VTTcv	Unigram	.729	.608	.789	.831
VTT	Bigram	.736	.617	.797	.836
VTT	Unigram	.733	.606	.789	.822

2.1 Sentence performance: Feature transforms and dimensionality reduction

The following charts show the F1 and MCC performance performance on bigram runs when feature transforms and dimensionality reductions are applied. Significant improvements (p<0.05, one-tailed test) compared to the same classifier applied to non-transformed data are indicated by stars.

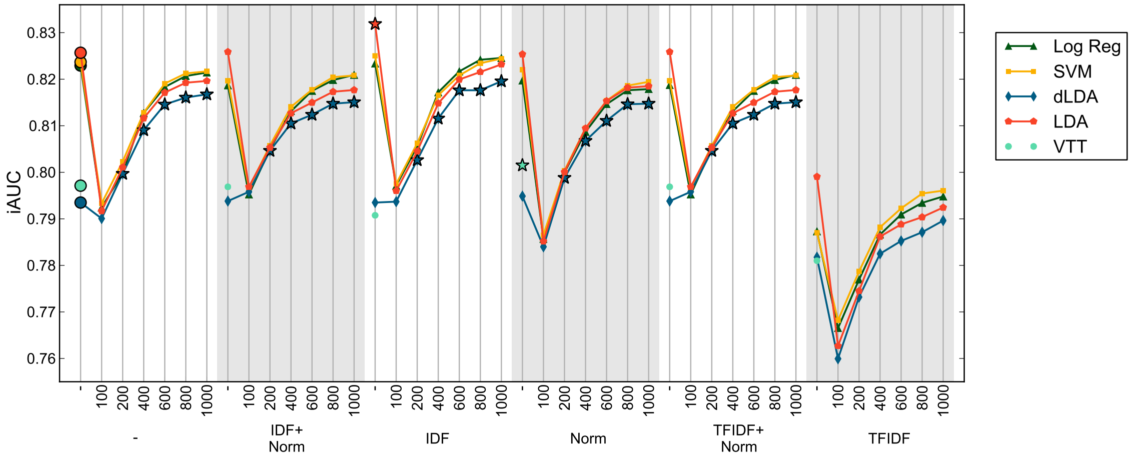

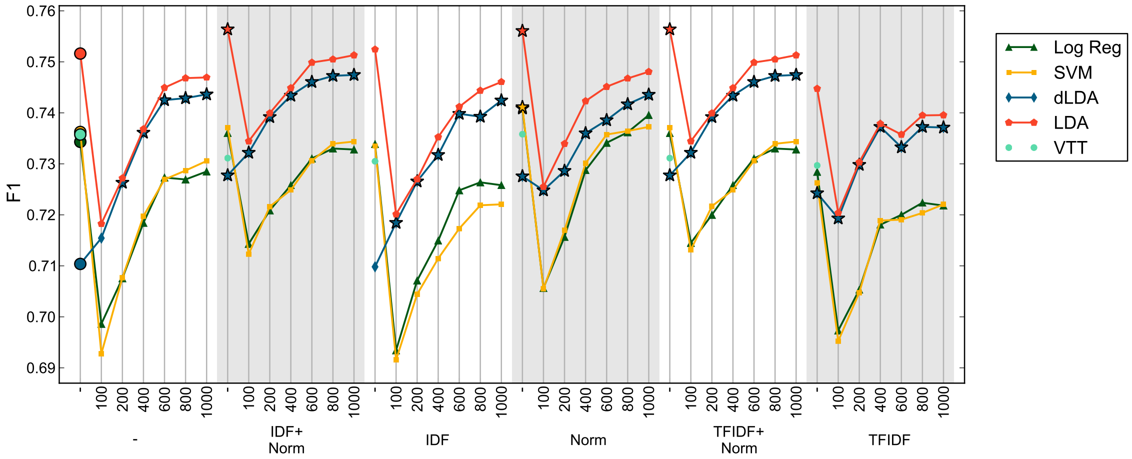

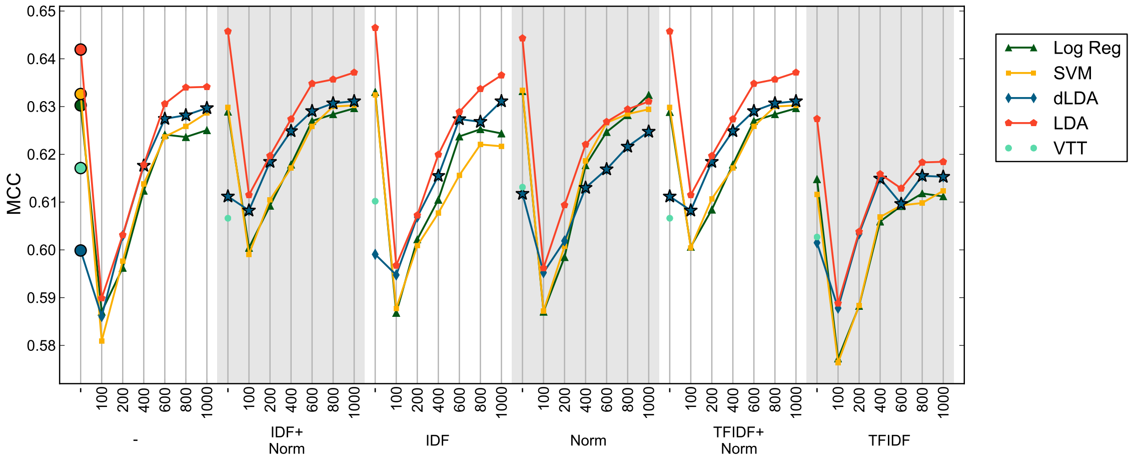

2.2 Sentence Performance: Most relevant and irrelevant features

We analyzed which features were most relevant and irrelevant for identifying evidence sentences using the same methodology as for abstracts (described in section 1.2). In the following figure, *RELEVANT FEATURES* includes any feature whose standardized coefficient was among the top 5 most positive standardized coefficients for any transform/classifier combination, while *IRRELEVANT FEATURES* includes any whose standardized coefficient was among the top 5 most negative standardized coefficients for any transform/classifier combination. Transforms are organized in the vertical columns, while classifiers are distinguished by color and marker style. For relevant (irrelevant) features, markers are positioned according to their rank (reverse rank) among the most positive (negative) features for a given classifier and transform combination.

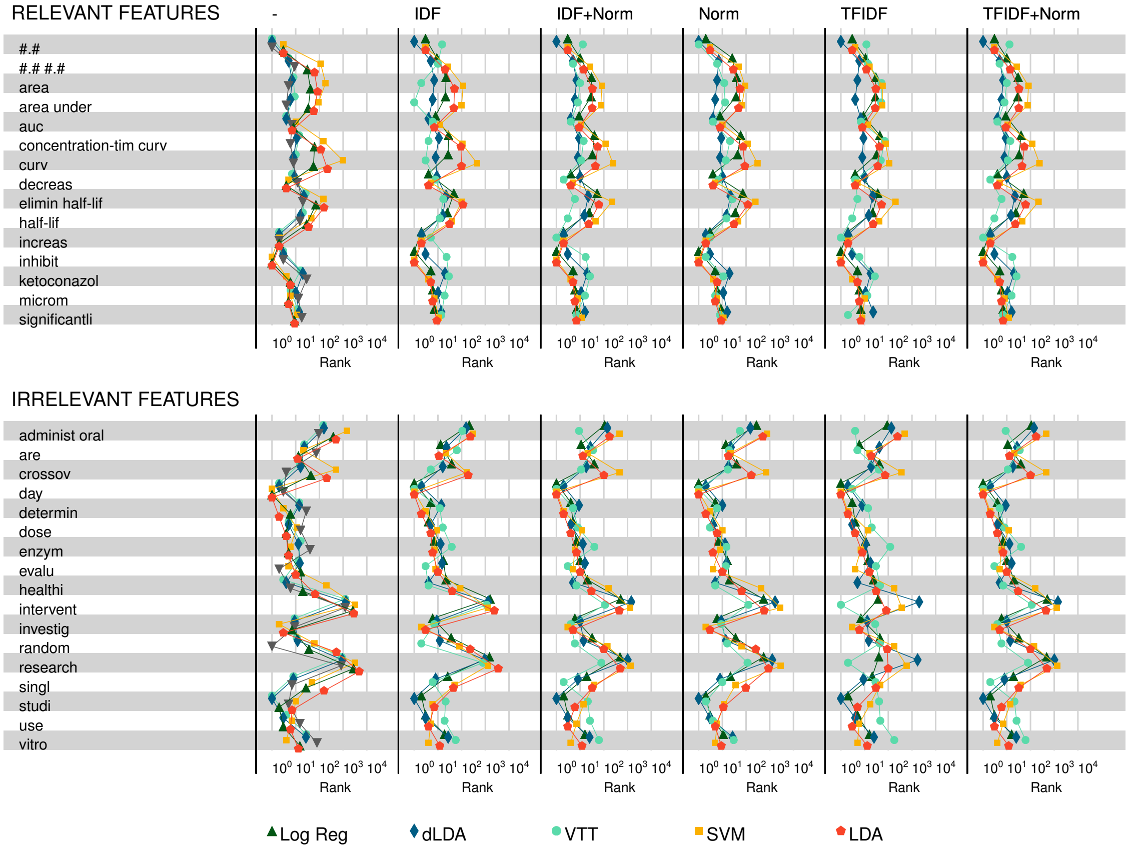

As in section 1.2, we perform principal component analysis of separating hyperplanes produced by different transforms and classifiers trained on the sentence corpus. Hyperplanes are generally grouped by classifier in this projection, with those corresponding to 'naive' classifiers that do not use feature covariances (VTT, dLDA, Naive Bayes) clustering separately from those that do (LDA, SVM, Logistic Regression).

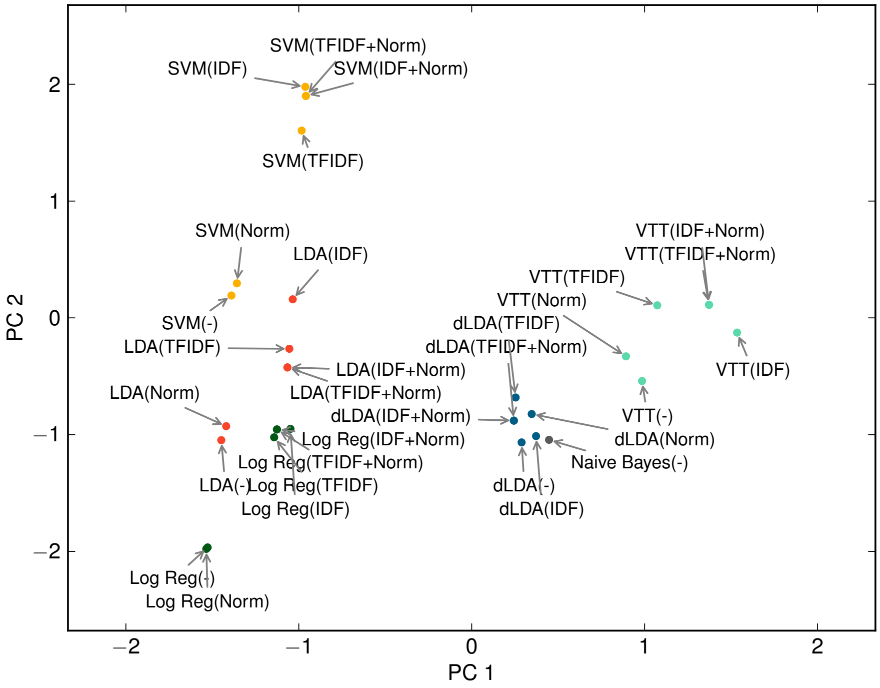

2.3 Sentence Performance: Impact of NER Features

The following figures plot the relative changes in F1 and MCC performance when including vs. not-including NER-derived features on non-transformed sentence bigram runs. Significant changes (p<0.05, two-tailed test) indicated by asterisks.

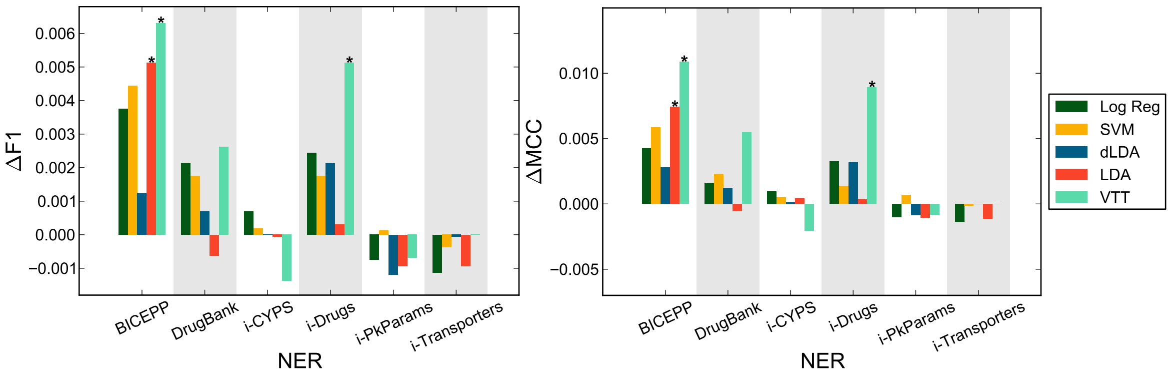

Supplement: S1 Text — Detailed performance measures on both abstract and sentence tasks, including when dimensionality reduction methods and NER and metadata features are used. In addition, the most relevant and irrelevant features for different classifiers and feature transform configuration are provided for both tasks. Feature weight coefficients for different classifiers are compared using PCA. (PDF) [file pone.0122199.s001.pdf]
